# Supplementary material for: Development and Application of a Physiologically Based Pharmacokinetic Model for Diclazuril in Broiler Chickens
Source: Animals (Basel). 2023 Apr 29;13(9):1512. doi: 10.3390/ani13091512 (PMC10177140; doi:10.3390/ani13091512)
Supplement: Supplementary file 1 [file animals-13-01512-s001.zip › animals-2336159-supplementary.pdf]

Supplementary Materials

Development and Application of a Physiologically Based Pharmacokinetic Model for Diclazuril in Broiler Chickens

Fang Yang, Mei Zhang, Yang-Guang Jin, Jun-Cheng Chen, Ming-Hui Duan, Yue Liu, Ze-En Li, Xing-Ping Li and Fan Yang *

College of Animal Science and Technology, Henan University of Science and Technology, Luoyang 471023, China

* Correspondence: yfscau@126.com

Contents

1. PBPK model code written in acsl language in acslXtreme software...3
2. **M-type code file used to calculate WT based on the MRL** 8
3. Supplementary figures 10
Figure S1. Sensitivity analysis results for those non-influential parameters on diclazuril concentrations in muscle...10
Figure S2. Prediction of the diclazuril concentrations (curves) and comparisons with the corresponding MRL (horizontal line) in plasma (a), lung (b), skin + fat (c), kidney (d), muscle (e), and liver (f) after 5 consecutive days of administering medicated feed containing 1 mg/kg of diclazuril.11
Figure S3. Prediction of the diclazuril concentrations (curves) and comparisons with the corresponding MRL (horizontal line) in plasma (a), lung (b), skin + fat (c), kidney (d), muscle (e), and liver (f) after 10 consecutive days of administering medicated feed containing 1 mg/kg of diclazuril. 12
Figure S4. Prediction of the diclazuril concentrations (curves) and comparisons with the corresponding MRL (horizontal line) in plasma (a), lung (b), skin + fat (c), kidney (d), muscle (e), and liver (f) after 15 consecutive days of administering medicated feed containing 1 mg/kg of diclazuril. 13
Figure S5. Prediction of the diclazuril concentrations (curves) and comparisons with the corresponding MRL (horizontal line) in plasma (a), lung (b), skin + fat (c), kidney (d), muscle (e), and liver (f) after 20 consecutive days of administering medicated feed containing 1 mg/kg of diclazuril. 14
Figure S6. Prediction of the diclazuril concentrations (curves) and comparisons with the corresponding MRL (horizontal line) in plasma (a), lung (b), skin + fat (c), kidney (d), muscle (e), and liver (f) after 5 consecutive days of administering medicated water containing 1 mg/kg of diclazuril. 15
Figure S7. Prediction of the diclazuril concentrations (curves) and comparisons with the corresponding MRL (horizontal line) in plasma (a), lung (b), skin + fat (c), kidney (d), muscle (e), and liver (f) after 10 consecutive days of administering medicated water containing 1 mg/kg of diclazuril. 16
Figure S8. Prediction of the diclazuril concentrations (curves) and comparisons with the corresponding MRL (horizontal line) in plasma (a), lung (b), skin + fat (c), kidney (d), muscle (e), and liver (f) after 15 consecutive days of administering medicated water containing 1 mg/kg of diclazuril. 17
Figure S9. Prediction of the diclazuril concentrations (curves) and comparisons with the corresponding MRL (horizontal line) in plasma (a), lung (b), skin + fat (c), kidney (d), muscle (e), and liver (f) after 20 consecutive days of administering medicated water containing 1 mg/kg of diclazuril. 18
Figure S11. Prediction of the diclazuril concentrations (curves) and comparisons with the corresponding MRL (horizontal line) in plasma (a), lung (b), skin + fat (c), kidney (d), muscle (e), and liver (f) after 9 consecutive days of administering medicated water containing 3 mg/kg of diclazuril. 20

1. PBPK model code written in acsl language in acslXtreme software

```
PROGRAM
! This model was developed by Prof. Yang to predict the residue of diclazuril in chicken after multiple doses through medicated water.
! The physiological and anatomical parameters in chickens were mainly derived from the review by Wang et al (2020).
! The specific information is as follows: Physiological parameter values for physiologically based pharmacokinetic models in food-producing animals. Part II: Chicken and turkey
    INITIAL
    ! code that is executed once at the beginning of a simulation run goes here
  !!! body weight and tissue weight
        constant bw=1.5              ! bodyweight, kg; 1.34 in Wen's paper, 1.52 kg in He's paper.
                                       ! The SD values could be found in the previous studies.
        constant vcmu=0.5712       ! Fraction of bw, muscle
                                       ! The SD value is 0.1473. Wang et al. 2020, Table 1.
        constant vcli=0.0214       ! Fraction of bw, liver
                                       ! The SD value is 0.0047. Wang et al. 2020, Table 1.
        constant vcki=0.0064       ! Fraction of bw, kidney
                                       ! The SD value is 0.001. Wang et al. 2020, Table 1.
        constant vcbl=0.0483       ! Fraction of bw, plasma
                                       ! The SD value is 0.0098. Wang et al. 2020, Table 1.
        constant vcfa=0.134        ! Fraction of bw, fat
                                       ! The SD value was not available, and assumed to be 0.0201.
        constant vclu=0.0071       ! Fraction of bw, lung
                                       ! The SD value is 0.001. Wang et al. 2020, Table 1.
        constant vcsk=0.1338       ! Fraction of bw, skin
                                       ! The SD value is 0.0282. Wang et al. 2020, Table 1.
        vcre=1-(vcmu+vcli+vcki+vcbl+vcfa+vclu+vcsk)    ! Fraction of bw, the virtual rest compartment.
                                       ! This value was calculated based on 1 minus the sum of all tissues.
                                       ! The SD value was not available for this value.
        !! tissue weight, kg, equal to L.
        vmu=vcmu*bw      ! muscle, kg
        vli=vcli*bw      ! liver, kg
        vki=vcki*bw      ! kidney, kg
        vbl=vcbl*bw      ! blood, L
        vfa=vcfa*bw      ! fat, kg
```

```
vlu=vclu*bw      ! lung, kg
vsk=vcsk*bw      ! skin, kg
vre=vcre*bw      ! the virtual rest compartment, kg
vab=vbl/3        ! arterial blood, L
vap=vab*(1-pcv)    ! arterial plasma, L
vvb=vbl*2/3        ! venous blood, L
vvp=vvb*(1-pcv)    ! venous plasma, L
vsf=vfa+vsk      ! skin+fat, kg
!!! blood flow and cardiac output (L/h/kg bw)
constant CO=9.88         ! cardiac output, L/h/kg bw.
                         ! the SD value is 2.07, Wang et al. 2020, Table 13.
QTOT=CO*bw               ! L/h, translate its unit from L/h/kg bw to L/h.
constant qcmu=0.0764 ! Fraction of QTOT, muscle;
                         ! Its SD value is 0.0114, Wang et al. 2020, Table 16.
constant qcli=0.2526 ! Fraction of QTOT, liver
                         ! Its SD value is 0.1163, Wang et al. 2020, Table 16.
constant qcki=0.2012 ! Fraction of QTOT, kidney
                         ! Its SD value is 0.1244, Wang et al. 2020, Table 16.
constant qcfa=0.1        ! Fraction of QTOT, fat
                         ! Its SD value is 0.04, Wang et al. 2020, Table 16.
constant qclu=1          ! Fraction of QTOT, lung
                         ! There was no SD value for this parameter.
constant qcsk=0.1505 ! Fraction of QTOT, skin
                         ! Its SD value is 0.0313, Wang et al. 2020, Table 16.
qcre=1-(qcmu+qcli+qcki+qcfa+qcsk) ! Fraction of QTOT, the virtual rest compartment
                         ! This value was calculated based on 1 minus the sum of all tissues, excluding lung.
                         ! The SD value was not available for this value.
!! blood flow, L/h
qmu=QTOT*qcmu    ! muscle
qli=QTOT*qcli    ! liver
qki=QTOT*qcki    ! kidney
qfa=QTOT*qcfa    ! fat
qlu=QTOT*qclu    ! lung, this value was equal to the cardiac output (QTOT).
qsk=QTOT*qcsk    ! skin
qre=QTOT*qcre    ! the virtual rest compartment
```

qsf=qfa+qsk ! skin + fat

!!! Partition coefficient (P) for diclazuril, unitless

constant pmu=0.1299 ! muscle, calculated based on area method (Gallo et al. 1987).

constant pki=0.6813 ! kidney, calculated based on area method (Gallo et al. 1987).

constant pli=0.9613 ! This value was optimized based on previous concentration-time data sets. More details could be found in the manuscript.

constant psf=0.0955 ! skin + fat, calculated based on area method (Gallo et al. 1987).

constant pre=1.2965 ! This value was optimized based on previous concentration-time data sets. More details could be found in the manuscript.

constant plu=0.5603 ! This value was optimized based on previous concentration-time data sets. More details could be found in the manuscript.

!!! doseing

constant dosewater=3 ! 3 mg/L, diclazuril in water, Wen (2007 and 2008)

constant dosefeed=0 ! 0.73 mg/kg, diclazuril in feed, Mortier et al. (2005)

constant waterperday=0.5 ! L, daily water intake per chicken

constant feedperday=1.1 ! kg, daily feed intake per chicken

dosingW=dosewater*waterperday*1000 ! ug per day

dosingF=dosefeed*feedperday*1000 ! ug per day

constant ka=0.1234 ! 1/h; the absorption rate constant

CONSTANT tlen = 12 ! h, the Length of oral exposure through water and feed was 12 and 16 (h/day), respectively

! This time is equal to the light time per day in the specific light regime.

CONSTANT tinterval = 24 ! Varied dependent on the exposure paradigm, 24 h

CONSTANT Dstart = 0 ! Initiation day of dosing (day). Please note the unite is day!

CONSTANT Dstop = 10 ! Termination day of dosing through water and feed was 9 and 11 days, respectively.

Tsim = TSTOP ! Tstop in hours

DS = Dstart*24 ! Initiation time point of the first dose (h)

Doff = (Dstop - Dstart)*24 ! The whole drug exposure duration (h)

TimeOn = Dstart*24

TimeOff = Dstop*24+tlen

!!! elimination from liver and with feces

constant clhe=0.00344 ! hepatic clearance (L/h/kg)

CCLhe=clhe*bw ! hepatic clearance (L/h)

```
   CONSTANT kgut=0.3838        ! excretion rate constant with feces (1/h)
   constant pcv=0.32              ! Hematocrit, unitless. This parameter was used to change the blood volume
to plasma volume.
     END ! INITIAL

     DYNAMIC
          ALGORITHM IALG = 2
          NSTEPS        NSTP = 10
          MAXTERVAL MAXT = 1.0e9
          MINTERVAL MINT = 1.0e-9
          CINTERVAL CINT = 1

          DERIVATIVE
!! multiple oral administration
    Exposure = PULSE(DS, Tsim, Doff)*PULSE(0,tinterval,tlen)
    RdoseFeed = dosingF/tlen*Exposure
    RdoseWater = dosingW/tlen*Exposure
    feedin=integ(RdoseFeed,0)
    waterin=integ(RdoseWater,0)
!! GI constant
    ragicon=RdoseFeed+RdoseWater-rabsp-routgut
    rabsp=ka*agicon
    absp=integ(rabsp,0)
    routgut=kgut*agicon
    outgut=integ(routgut,0)
    agicon=integ(ragicon,0)
!! liver
    rali=rabsp+qli*(cap-cli/pli)-routli
    routli=CCLhe*cli/pli
    ali=integ(rali,0)
    cli=ali/vli          ! ug/kg
    outli=integ(routli,0)
!! kideny
    raki=qki*(cap-cki/pki)
    aki=integ(raki,0)
    cki=aki/vki
```

```
!! skin+fat
     rasf=qsf*(cap-csf/psf)
     asf=integ(rasf,0)
     csf=asf/vsf
!! muscle
     ramu=qmu*(cap-cmu/pmu)
     amu=integ(ramu,0)
     cmu=amu/vmu
!! rest
     rare=qre*(cap-cre/pre)
     are=integ(rare,0)
     cre=are/vre
!! lung
     ralu=qlu*(cvp-clu/plu)
     alu=integ(ralu,0)
     clu=alu/vlu
!! arterial plasma
     raap=qlu*(clu/plu-cap)
     aap=integ(raap,0)
     cap=aap/vap
!! venous plasma
     ravp=-qlu*cvp+cmu/pmu*qmu+csf/psf*qsf+cki/pki*qki+cli/pli*qli+cre/pre*qre
     avp=integ(ravp,0)
     cvp=avp/vvp

!!!! check mass balance
   MB=feedin+waterin-outgut-(avp+aap+alu+are+amu+asf+aki+ali+agicon)-outli
          END ! DERIVATIVE

     !!!! Calculation of Absolute Bioavailability (AB)
   ABfeed=absp/feedin*100          !! %
   ABwater=absp/waterin*100        !! %
          CONSTANT TSTOP = 700.0
          TERMT (T .GE. TSTOP, 'checked on communication interval: REACHED TSTOP')
     END ! DYNAMIC
END ! PROGRAM
```

2. **M-type code file used to calculate WT based on the MRL**

```
MRLmu=500;          % MRL for diclazuril in muscle, ug/kg
MRLsf=1000;       % MRL for diclazuril in skin+fat, ug/kg
MRLli=3000;       % MRL for diclazuril in liver, ug/kg
MRLki=2000;       % MRL for diclazuril in kidney, ug/kg

maxt=max(_t);
time=[0:4:maxt];
time=flipud(time);
nn=length(time);
one=ones(1,nn);
li=one*MRLli;
ki=one*MRLki;
mu=one*MRLmu;
sf=one*MRLsf;
li=flipud(li);
ki=flipud(ki);
mu=flipud(mu);
sf=flipud(sf);

plot(time,li,_t,__cli_th__);
plot(time,ki,_t,__cki_th__);
plot(time,mu,_t,__cmu_th__);
plot(time,sf,_t,__csf_th__);

ta=flipud(_t);
cli=flipud(__cli_th__);
cki=flipud(__cki_th__);
cmu=flipud(__cmu_th__);
csf=flipud(__csf_th__);
```

```
for b=1:numIts;
liCH=find(cli(:,b)>MRLli);
if isempty (liCH)
wtliCH(b)=0;
else
firli=liCH(1)-1;
wtliCH(b)=ta(firli);
end
kiCH=find(cki(:,b)>MRLki);
if isempty (kiCH)
wtkiCH(b)=0;
else
firki=kiCH(1)-1;
wtkiCH(b)=ta(firki);
end
muCH=find(cmu(:,b)>MRLmu);
if isempty (muCH)
wtmuCH(b)=0;
else
firmu=muCH(1)-1;
wtmuCH(b)=ta(firmu);
end
sfCH=find(csf(:,b)>MRLsf);
if isempty (sfCH)
wtsfCH(b)=0;
else
firsf=sfCH(1)-1;
wtsfCH(b)=ta(firsf);
end
end
plot(wtliCH)
```

```
plot(wtkiCH)
plot(wtmuCH)
plot(wtsfCH)
```

3. Supplementary figures

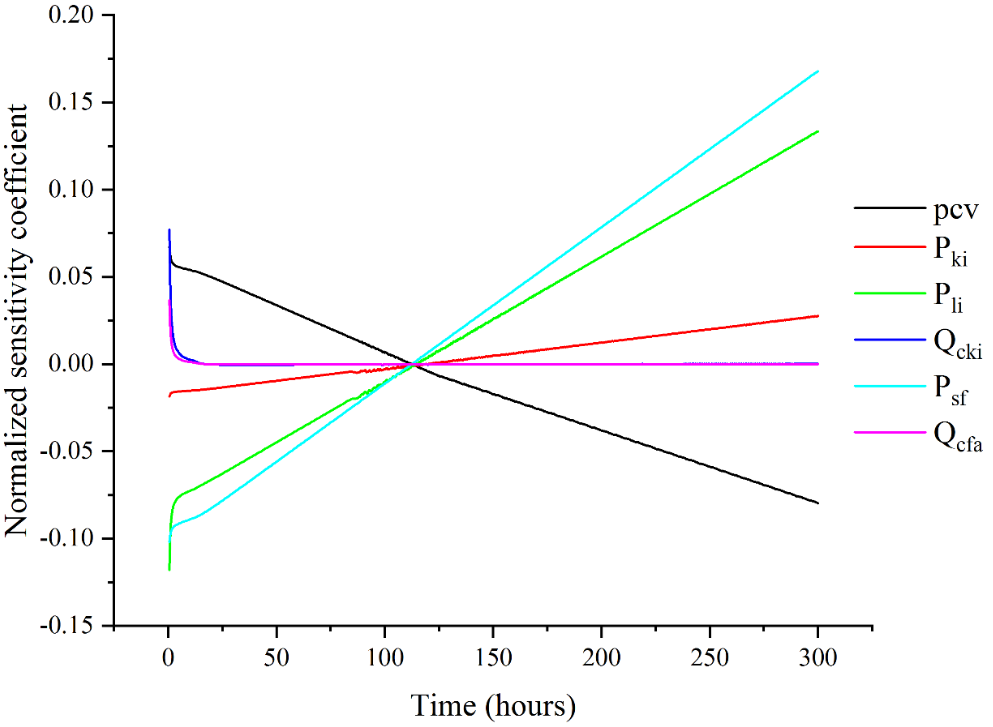

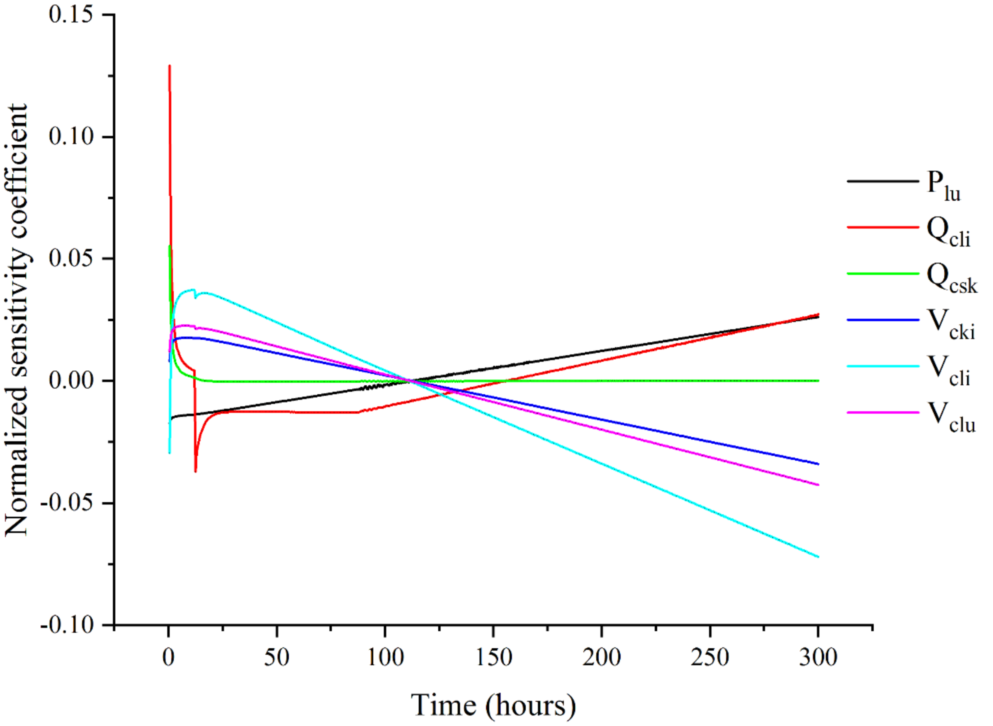

Figure S1. Sensitivity analysis results for those non-influential parameters on diclazuril concentrations in muscle.

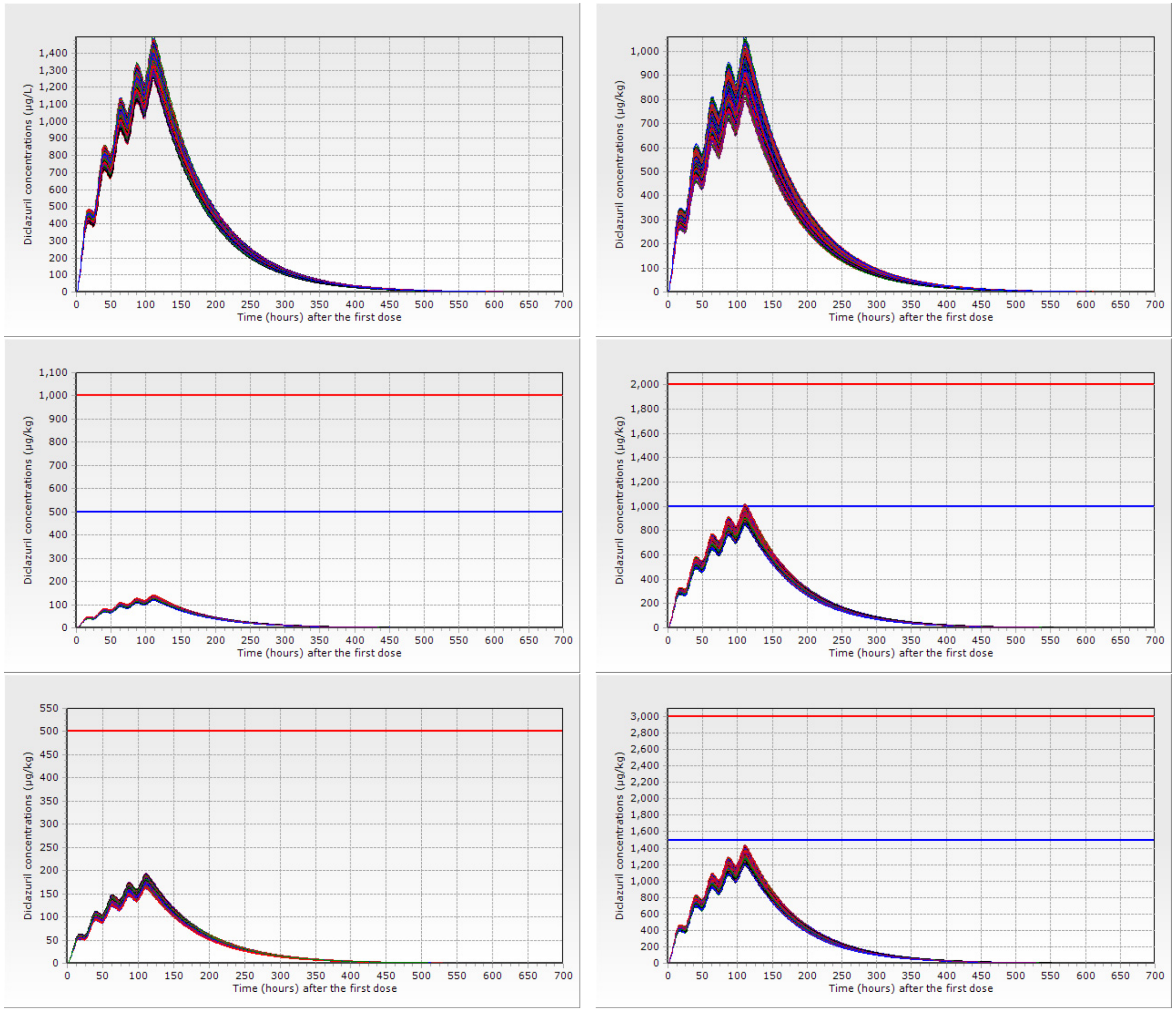

Figure S2. Prediction of the diclazuril concentrations (curves) and comparisons with the corresponding MRL (horizontal line) in plasma (a), lung (b), skin + fat (c), kidney (d), muscle (e), and liver (f) after 5 consecutive days of administering medicated feed containing 1 mg/kg of diclazuril. In muscle, China and Europe shared the same MRL value (500 µg/kg), which was represented by a red line. In the other three tissues, the red line is the Chinese value, and the blue line is the European value.

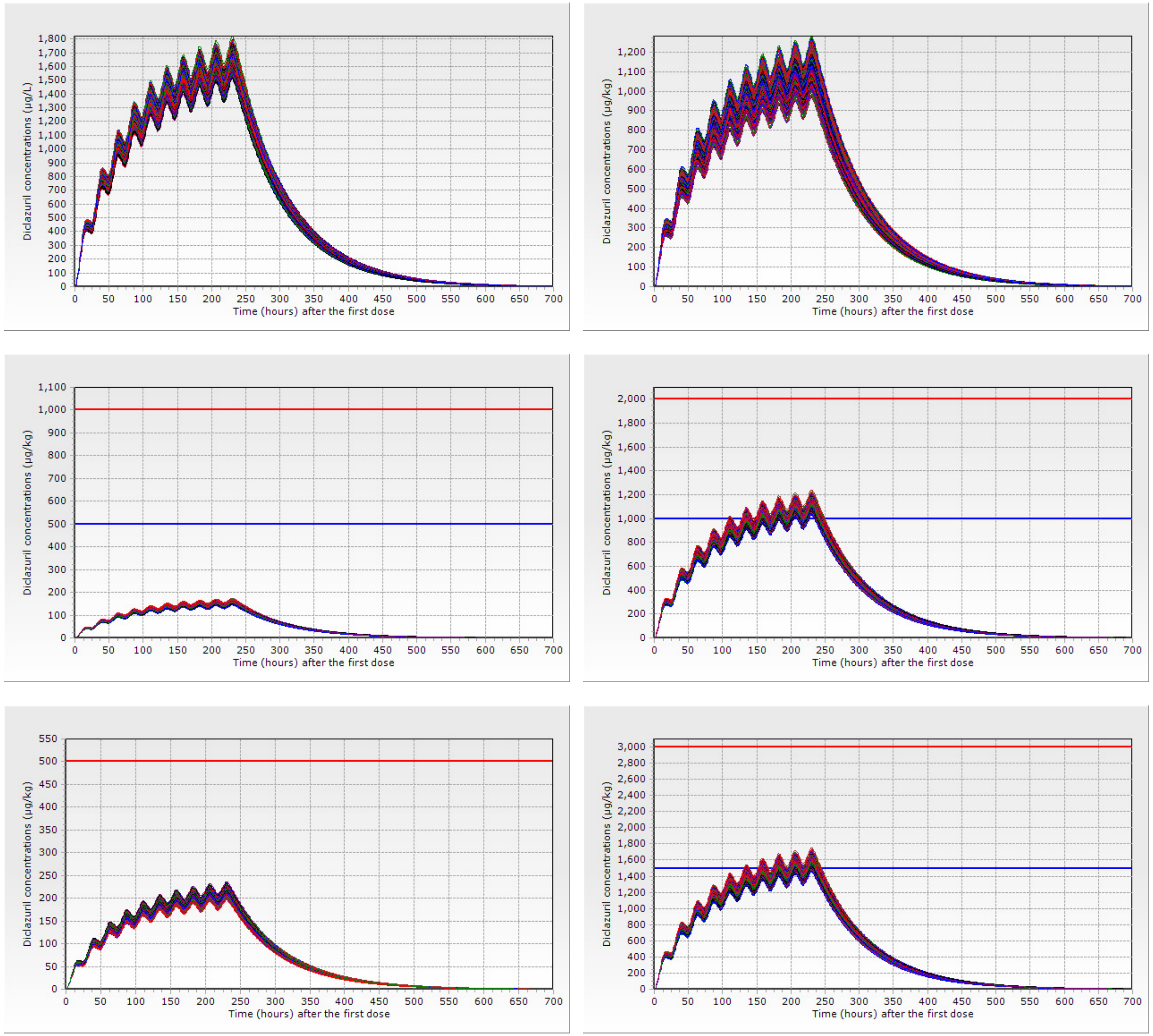

Figure S3. Prediction of the diclazuril concentrations (curves) and comparisons with the corresponding MRL (horizontal line) in plasma (a), lung (b), skin + fat (c), kidney (d), muscle (e), and liver (f) after 10 consecutive days of administering medicated feed containing 1 mg/kg of diclazuril.

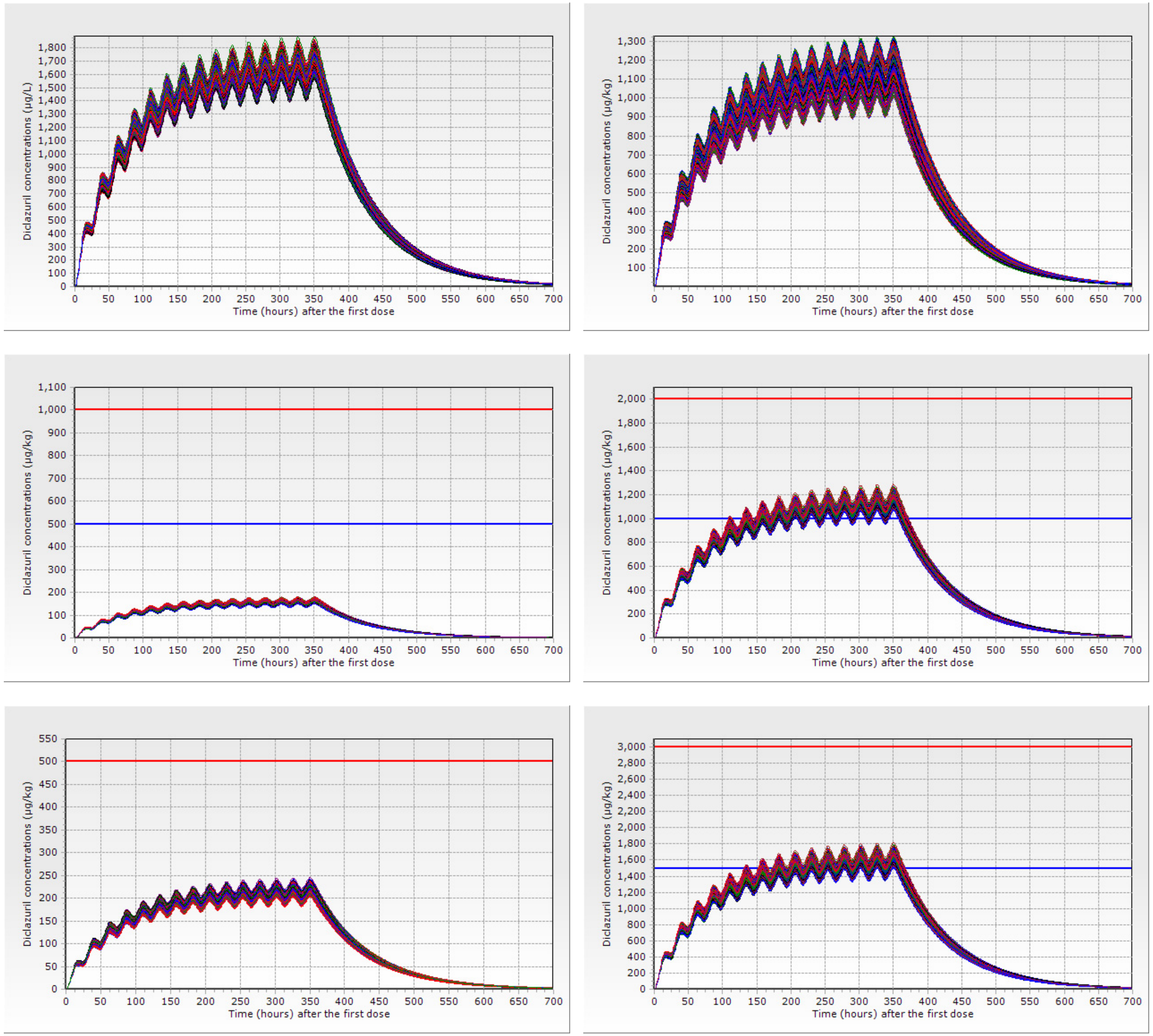

Figure S4. Prediction of the diclazuril concentrations (curves) and comparisons with the corresponding MRL (horizontal line) in plasma (a), lung (b), skin + fat (c), kidney (d), muscle (e), and liver (f) after 15 consecutive days of administering medicated feed containing 1 mg/kg of diclazuril.

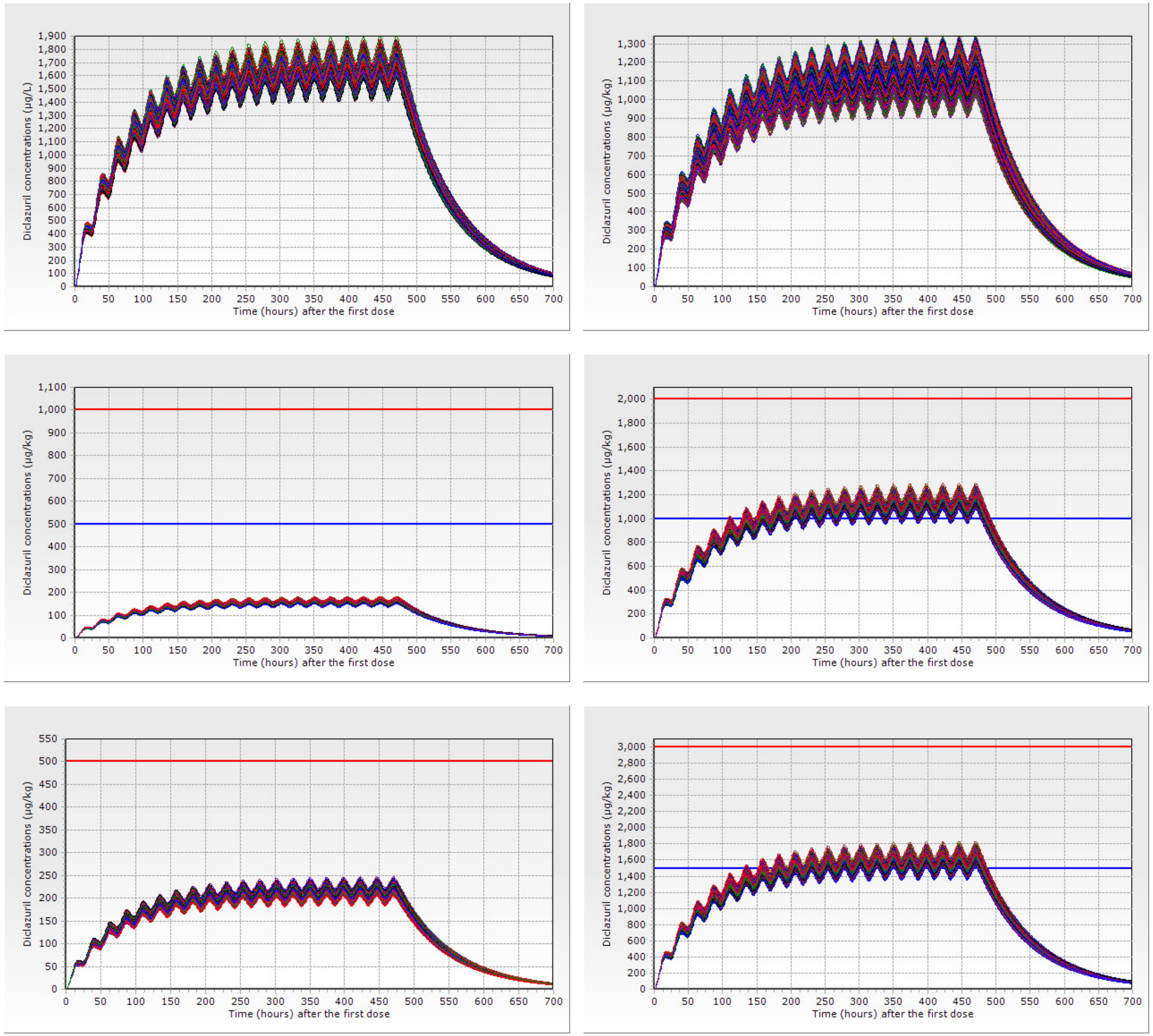

Figure S5. Prediction of the diclazuril concentrations (curves) and comparisons with the corresponding MRL (horizontal line) in plasma (a), lung (b), skin + fat (c), kidney (d), muscle (e), and liver (f) after 20 consecutive days of administering medicated feed containing 1 mg/kg of diclazuril.

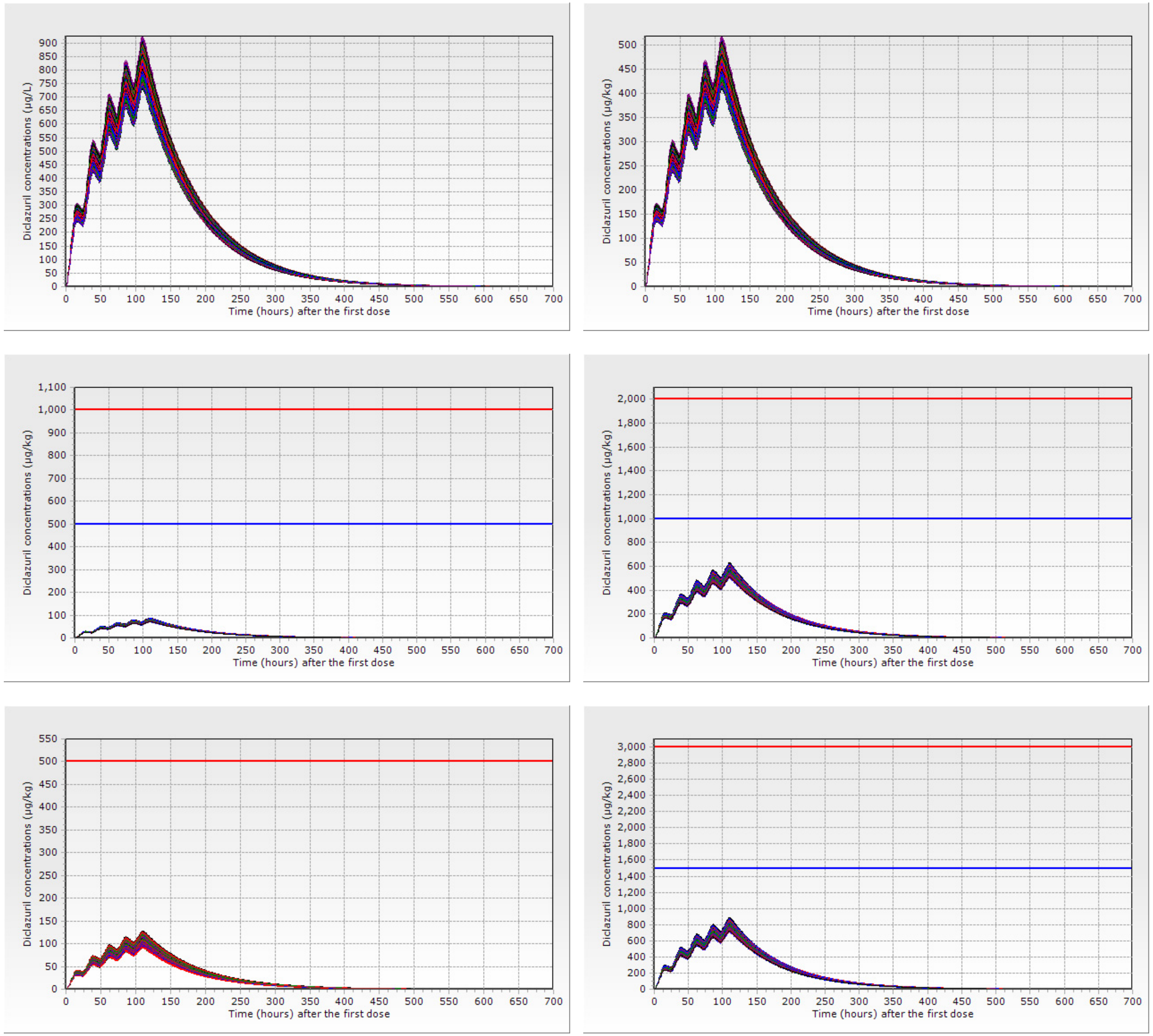

Figure S6. Prediction of the diclazuril concentrations (curves) and comparisons with the corresponding MRL (horizontal line) in plasma (a), lung (b), skin + fat (c), kidney (d), muscle (e), and liver (f) after 5 consecutive days of administering medicated water containing 1 mg/L of diclazuril.

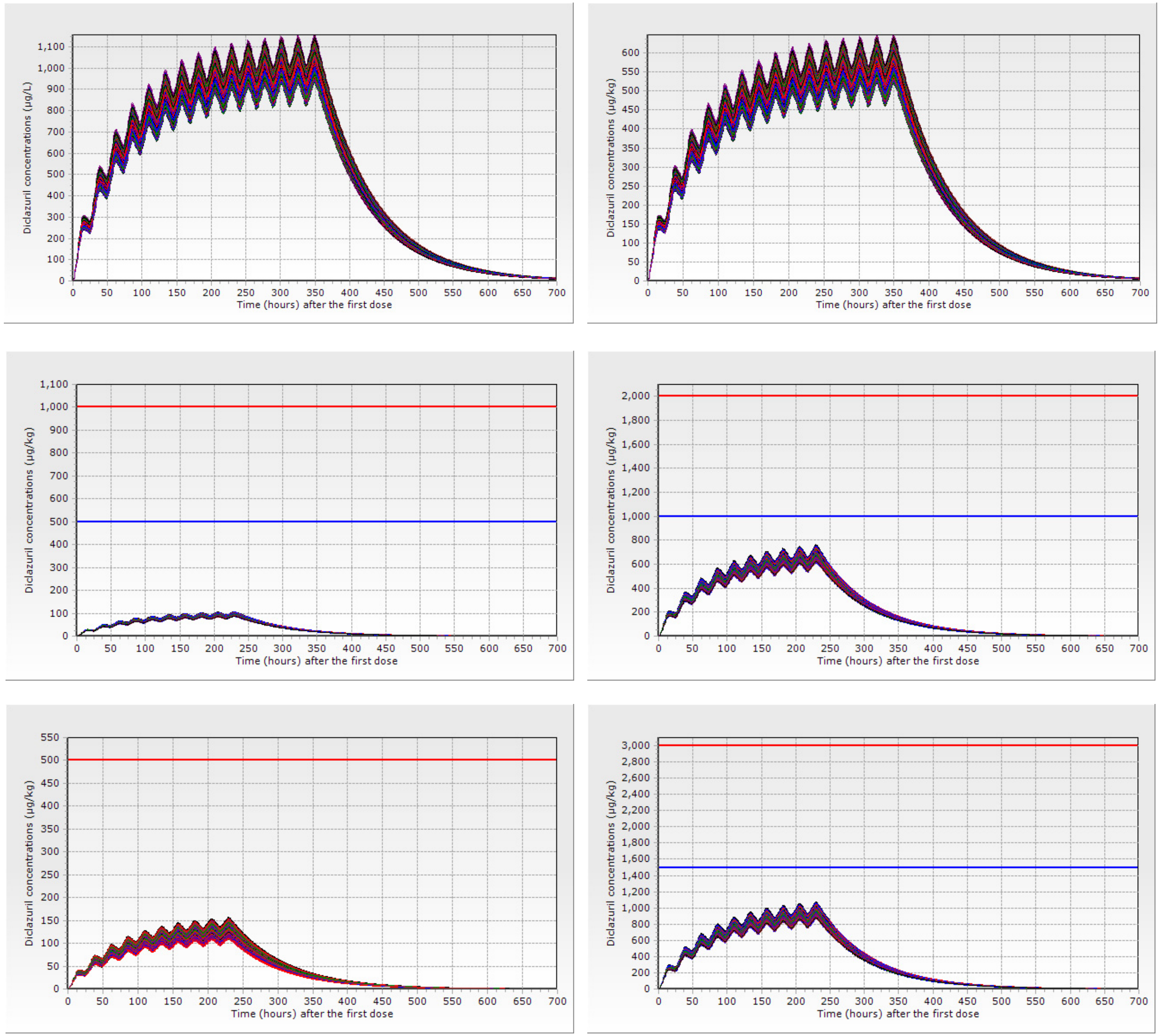

Figure S7. Prediction of the diclazuril concentrations (curves) and comparisons with the corresponding MRL (horizontal line) in plasma (a), lung (b), skin + fat (c), kidney (d), muscle (e), and liver (f) after 10 consecutive days of administering medicated water containing 1 mg/L of diclazuril.

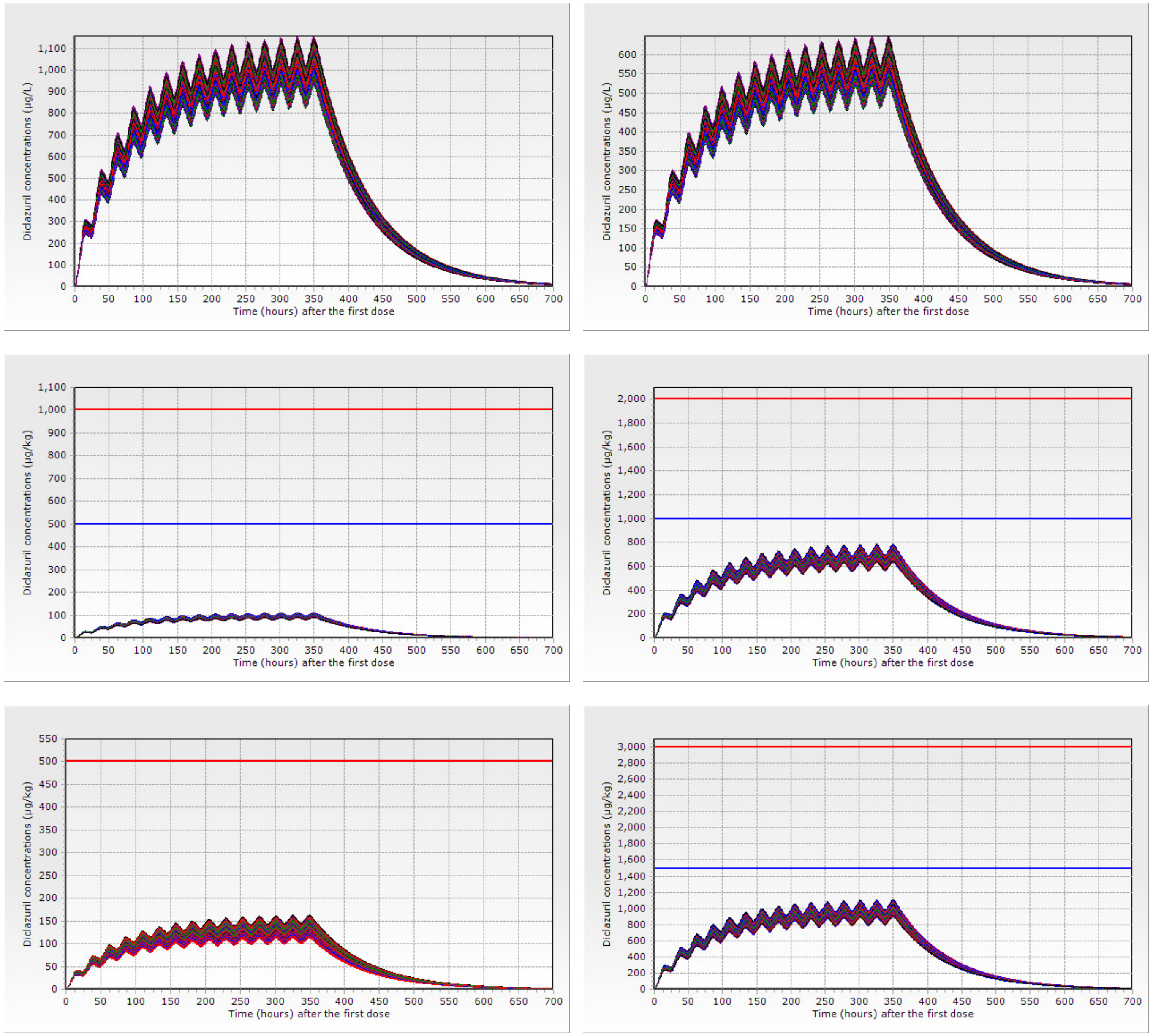

Figure S8. Prediction of the diclazuril concentrations (curves) and comparisons with the corresponding MRL (horizontal line) in plasma (a), lung (b), skin + fat (c), kidney (d), muscle (e), and liver (f) after 15 consecutive days of administering medicated water containing 1 mg/L of diclazuril.

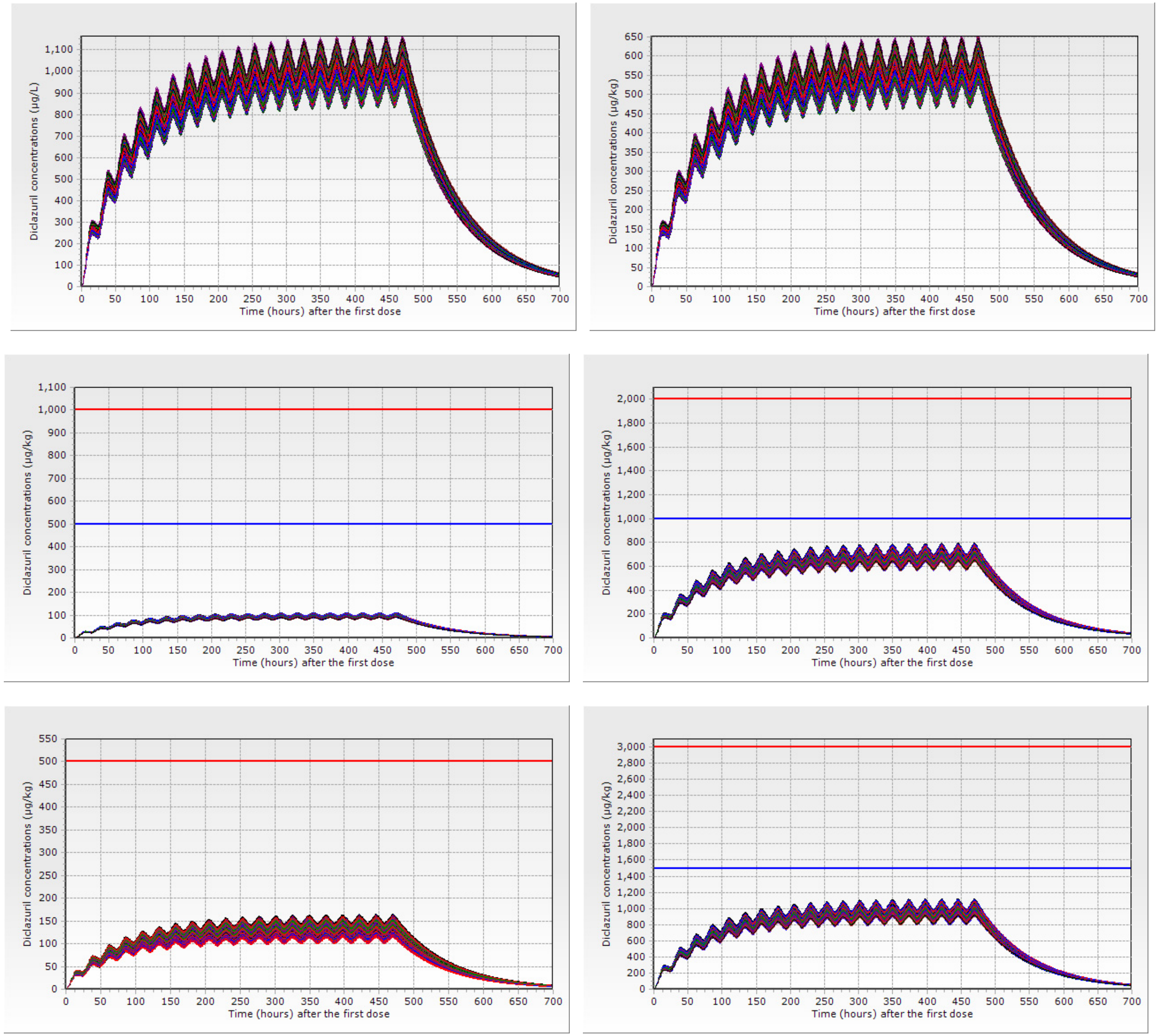

Figure S9. Prediction of the diclazuril concentrations (curves) and comparisons with the corresponding MRL (horizontal line) in plasma (a), lung (b), skin + fat (c), kidney (d), muscle (e), and liver (f) after 20 consecutive days of administering medicated water containing 1 mg/L of diclazuril.

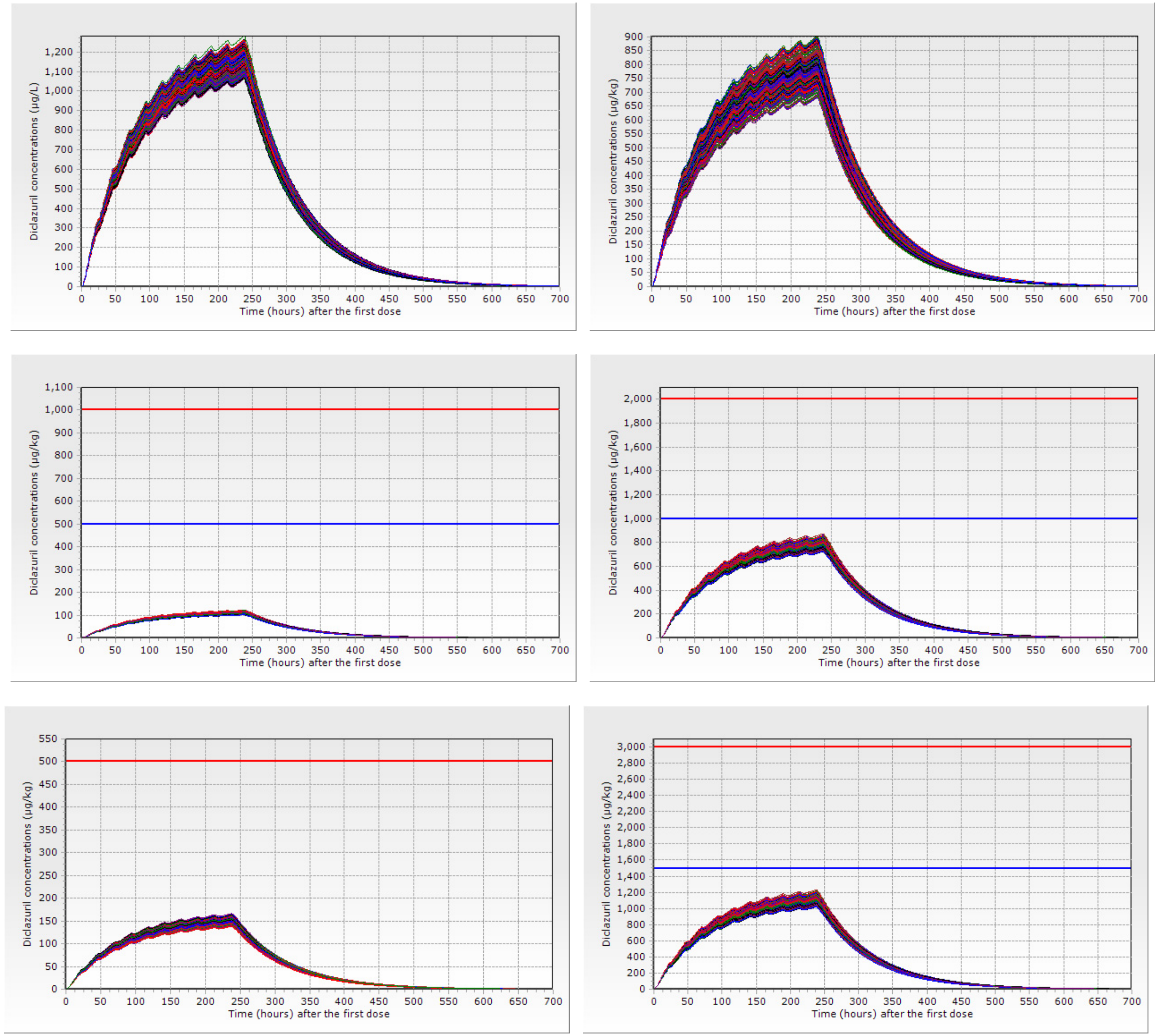

Figure S10. Prediction of the diclazuril concentrations (curves) and comparisons with the corresponding MRL (horizontal line) in plasma (a), lung (b), skin + fat (c), kidney (d), muscle (e), and liver (f) after 10 consecutive days of administering medicated feed containing 730 µg/kg of diclazuril.

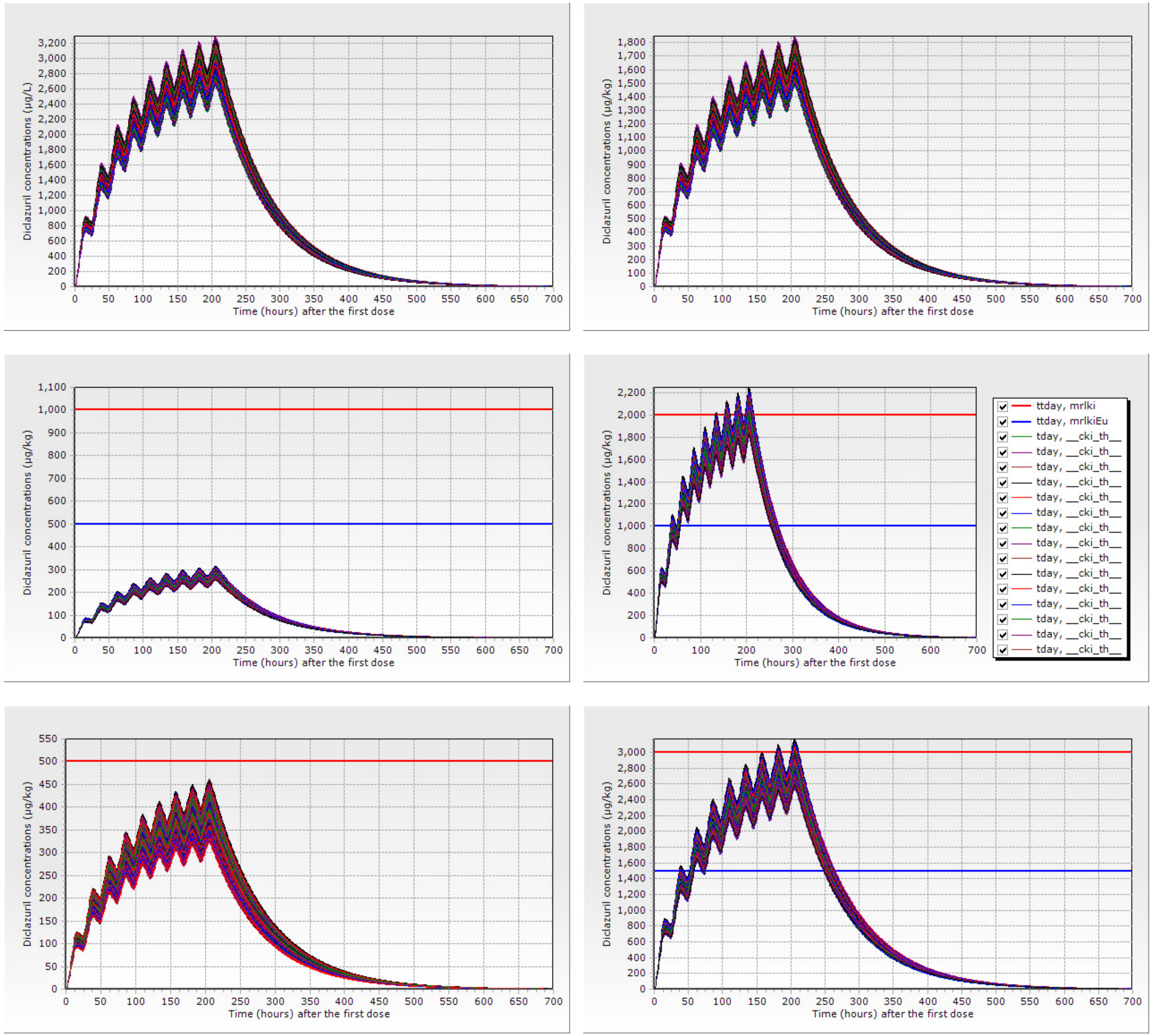

Figure S11. Prediction of the diclazuril concentrations (curves) and comparisons with the corresponding MRL (horizontal line) in plasma (a), lung (b), skin + fat (c), kidney (d), muscle (e), and liver (f) after 9 consecutive days of administering medicated water containing 3 mg/L of diclazuril.
